# Hsa-miR-590-5p Interaction with SMAD3 Transcript Supports Its Regulatory Effect on The TGFβ Signaling Pathway 

**DOI:** 10.22074/cellj.2016.3981

**Published:** 2016-04-04

**Authors:** Meisam Jafarzadeh, Bahram M. Soltani

**Affiliations:** Department of Molecular Genetics, Faculty of Biological Sciences, Tarbiat Modares University, Tehran, Iran

**Keywords:** Hsa-miR-590-5p, SMAD3, TGFβ

## Abstract

**Objective:**

SMAD proteins are the core players of the transforming growth factor-beta (TGFβ) signaling pathway, a pathway which is involved in cell proliferation, differentiation and migration. On the other hand, hsa-miRNA-590-5p (miR-590-5p) is known to have a negative regulatory effect on TGFβ signaling pathway receptors. Since, RNAhybrid analy- sis suggested SMAD3 as a bona fide target gene for miR-590, we intended to investigate the effect of miR-590-5p on SMAD3 transcription.

**Materials and Methods:**

In this experimental study, miR-590-5p was overexpressed in different cell lines and its increased expression was detected through quantitative reverse transcription-polymerase chain reaction (RT-qPCR). Western blot analysis was then used to investigate the effect of miR-590-5p overexpression on SMAD3 protein level. Next, the direct interaction of miR-590-5p with the 3´-UTR sequence of SMAD3 transcript was investigated using the dual luciferase assay. Finally, flow cytometery was used to inves- tigate the effect of miR-590-5p overexpression on cell cycle progression in HeLa and SW480 cell lines.

**Results:**

miR-590-5p was overexpressed in the SW480 cell line and its overexpression resulted in significant reduction of the SMAD3 protein level. Consistently, direct interaction of miR-590-5p with 3´-UTR sequence of SMAD3 was detected. Finally, miR-590-5p over- expression did not show a significant effect on cell cycle progression of Hela and SW480 cell lines.

**Conclusion:**

Consistent with previous reports about the negative regulatory effect of miR-590 on TGFβ receptors, our data suggest that miR-590-5p also attenuates the TGFβ signaling pathway through down-regulation of SMAD3.

## Introduction

The transforming growth factor-beta (TGFβ) superfamily which includes TGFβ, bone morphogenic proteins (BMPs) and activin/inhibin plays critical roles in embryonic development and adult tissue homeostasis by affecting cell proliferation, differentiation and migration (1,2). The canonical TGFβ/BMP signaling is mediated through a simple linear cascade which is initiated by the activation of TGFβ type I and II receptors in a ligand binding manner (3). The receptor-activated SMADs (R-SMADs) including SMAD2 and SMAD3 are then phosphorylated, with this followed by R-SMAD and common mediator SMAD (co-SMAD) heterodimer formation (4). The resulting complexes then re-locate to the nucleus where they regulate transcription of their target genes together with numerous other factors (3,4). The SMAD proteins are thus the core players of the TGFβ/BMP signaling pathway. These proteins bind to the 12-O-tetradecanoyl-13-acetate (TPA) responsive element (TRE) in the promoter of many genes, especially those that have a major role in cancer development (5,8). Regulation of these signaling molecules is therefore one of the important steps in TGFβ signaling pathway. 

MicroRNAs (miRNA) are small non-coding RNAs which post-transcriptionally regulate the expression of a large number of protein-coding genes (9,10) including the genes involved in proliferation, differentiation, apoptosis and tumor genesis (11,14). miR-590 has been suggested to be a regulator of TGFβ signaling pathway which exerts its effect through targeting TGFβ receptors (TGFβRs) during cardiac cell development (15). Here, we investigated and showed the effect of miR-590-5p on SMAD3 transcription as another major TGFβ signaling component. 

## Materials and Methods

### Bioinformatics

Prediction of miRNA targets was carried out by RNAhybrid (http://bibiserv.techfak.uni-bielefeld. de/rnahybrid), a tool for finding the minimum free energy of hybridization of a miRNA. 

### Cell culture

In this experimental study, human cell lines (HeLa, HEK293t and SW480) and cardiospherederived cells (CDCs) were cultured in Hepesbuffered Dulbecco’s modified Eagle’s medium (H-DMEM) and Iscove’s Modified Dulbecco’s Medium (IMDM) complete medium respectively, both supplemented with 10% fetal bovine serum (FBS, Gibco, USA) and 1% penicillin streptomycin (PS, Gibco, USA), and incubated at 37˚C with 5% CO_2_. HeLa, HEK293t and SW480 cell lines were bought from Pasteur Institute of Iran and human CDC were obtained from Royan Stem Cell Bank (national code number RSCB0180). Permission was obtained individually from the patients as source of cells using standard informed consent. 

### Transfection

For miRNA overexpression analysis, HeLa and SW480 cell lines were cultured in a 24-well plate and were allowed to adhere for 24 hours. Cells were then transiently transfected with pLenti-hsa-miR-590 and pLenti-blank expression vectors using Lipofectamine 2000 (Invitrogen, USA) and incomplete media lacking FBS and PS. Transfected cells were cultured for 6 hours, then culture media (incomplete) were replaced with complete media. Thirty hours after transfection, green fluorescent protein (GFP) expression was visually examined by fluorescent microscopy (Nikon eclipses Te2000-s). Finally, cells were harvested 48 hours after transfection and RNA extracted using Trizol (Invitrogen, USA). 

For the luciferase assay, Hek293t cell line was co-transfected with SMAD3 3´-UTR luciferase reporter construct (psiCHECK-2) and pLenti-III-miR590-GFP in a 96-well plate according to the manufacturer’s instructions. Dual luciferase assay was performed 48 hours after transfection. In order to correct vector-dependent unspecific effects, relative reporter activity was normalized to the empty vector co-transfected with the miRNA. Experiments were done in triplicate. 

### RNA extraction and quantitative reverse transcription-polymerase chain reaction

Total RNA was extracted from the SW480 cell line using Trizol according to the manufacturer’s protocol. The RNA quality and yield of extraction was analyzed by agarose gel electrophoresis (BioER, China) and spectrophotometry (Gene Quest, England) respectively. In order to remove any genomic traces, DNaseI treatment (Takara, Japan) was performed prior to cDNA synthesis at 37˚C for 30 minutes followed by heat and Ethylenediamine tetra acetic acid (EDTA, Sigma, USA) inactivation. cDNA was then synthesized by PrimeScript II reverse transcriptase (Takara, Japan) according to the manufacturer’s protocol. For miRNA detection, polyA tail was added to the 3´ end of RNAs before cDNA synthesis by using polyA polymerase (Takara, Japan) according to the manufacturer’s protocol. The anchored oligodT (5´-GCGTCGACTAGTACAACTCAAGGTTCTTCCAGTCACGACGTTTTTTTTTTTTTTTTTT- 3´) was used for cDNA synthesis. Real-time quantitative PCR was performed using standard protocols on an ABI PRISM 7500 instrument (Applied Biosystems, USA). The following primers were used for real-time PCR: miR-590-5p forward 5´-GAGCTTATTCATAAAAGTG-3´, U48 forward 5´-TGACCCCAGGTAACTCTGAGTGTGT-3´ and the Universal primer: 5´-AACTCAAGGTTCTTCCAGTCACG-3´. Output data were analyzed by GraphPad Prism. U48 SNO-RNA expression levels were used as internal control for normalization of miR-590 expression.

### Luciferase assay

In order to investigate direct interaction of miR-590 with SMAD3 3´UTR, Hek293t cells were co-transfected using psi-check2-SMAD3-3´-UTR and pLenti-hsa-miR-590-GFP. Psi-check2 reporter construct contained the Renilla luciferase gene just upstream of the SMAD3 3´-UTR and an independent firefly luciferase gene as an internal control for normalization. Forty-eight hours after co-transfection of both vectors into Hek293t cells, dual luciferase assay was performed using DualGlo (Promega, USA). The ratio of Renilla to firefly luciferase was taken and normalized relative to the cells transfected only by the psi-check2-SMAD33´-UTR vector. 

### Western blotting

Soluble proteins were extracted from CDC and subjected to electrophoresis on 12% Sodium dodecyl sulfate-polyacrylamide (Sigma, USA) gels, and then transferred to polyvinylidene fluoride (PVDF, Thermo Scientific, USA) membranes. This followed by 2 hours blocking by 5% milk (Sigma, USA) in tris-buffered saline containing 0.1% Tween (TBST, Bio basic, Canada) at RT and then incubated overnight at 4˚C with primary antibodies, SMAD3 (1:1000, Abcam, USA) and β-actin (1:2000, Santa Cruz Biotechnology, USA). Sheep anti-rabbit IgG-HRP secondary antibodies (1:1000, Santa Cruz Biotechnology, USA) were used on the second day for 1 hour at RT. Actin protein was used as loading control. The western blots were visualized with an ECL reaction kit (Beyotime, China), recorded on a Canon EOS 60D and quantitated using TotalLab Quant. 

### Cell cycle analysis

Forty-eight hours after transfection of hsa-miR-590 in HeLa and SW480 cell lines, cells were harvested and centrifuged at 1,200 rpm for 5 minutes and washed twice in PBS. Next, 1 ml ice-cold 70% ethanol (Bidestan, Iran) was added and cells were fixed in ethanol solution for at least 30 minutes. For each sample, 500 μl propidium iodide (PI, Sigma, USA) staining solution was added and samples were incubated for 30 minutes at room temperature. Number of cells in each cell cycle phase was determined by a Fluorescence Activated Cell Sorting (FACS) Calibur flow cytometer and analyzed using Cell Quest software (BD Biosciences, USA). 

## Results

### Bioinformatics analysis

**Fig.1:**
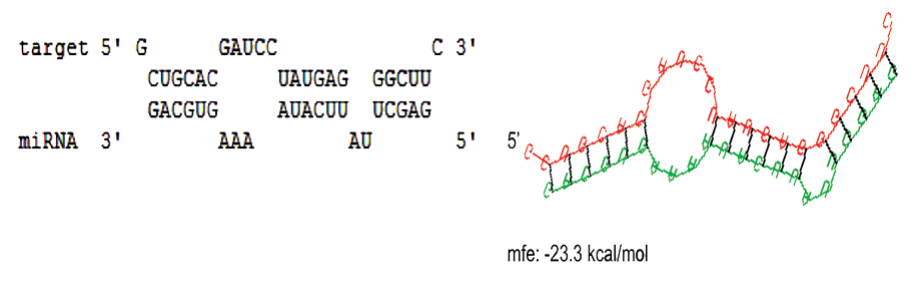
Pairing status of hsa-miR-590-5p with its predicted microRNA recognition element (MRE) located in the 3´-UTR sequence of SMAD3. Pairing of specific nucleotides between hsa-miR-590 and MRE are shown on the left. The secondary structure of hsa-miR-590 (green) and MRE (red) pairing is shown on the right. mfe: Minimum free energy of pairing.

According to RNAhybrid analysis, SMAD3 was introduced as a bona fide target of miR-590-5p showing near to perfect complementarity between them (Fig.1). Predicted binding site of miR-590 was the 1666^th^nucleotide of the transcript located in the 3´-UTR of SMAD3. Binding energy of miR-590-5p and its complementary target sequence in SMAD3 was -23.3 kcal/mol, indicating high affinity and stable binding between them. 

### Overexpression of miR-590-5-p

**Fig.2:**
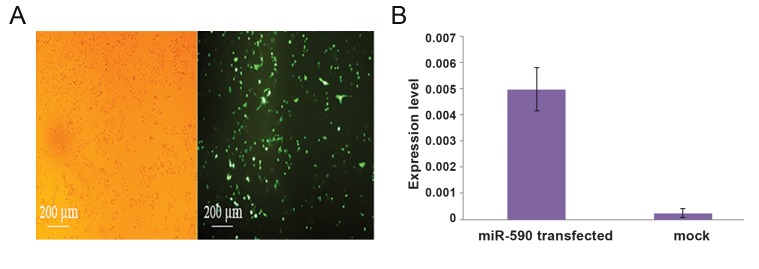
Overexpression of hsa-miR-590-5p in SW480 and CDC cells. A. GFP expression indicating successful transfection of SW480 cell line using pLenti-III recombinant vector resulting in hsa-miR-590-5p overexpression and B. RT-qPCR result showed more than 25-fold increased hsa-miR-590-5p expression following transfection of the CDC cells with pLenti-III recombinant vector compared with cells transfected with the empty vector (mock). CDC; Cardiosphere-derived cell, GFP; Green fluorescent protein and RT-qPCR; Quantitative reverse transcription-polymerase chain reaction.

Transfection of the pLenti-hsa-miR-590-5p expression vector into the CDC cell line showed about 70% transfection efficiency through GFP expression visualization (Fig.2A). Real-time PCR analysis confirmed the accuracy of transfection and overexpression of miR-590-5p (>25-fold, Fig.2B). 

### Down-regualtion of SMAD3 following miR-590-5p overexpression

**Fig.3:**
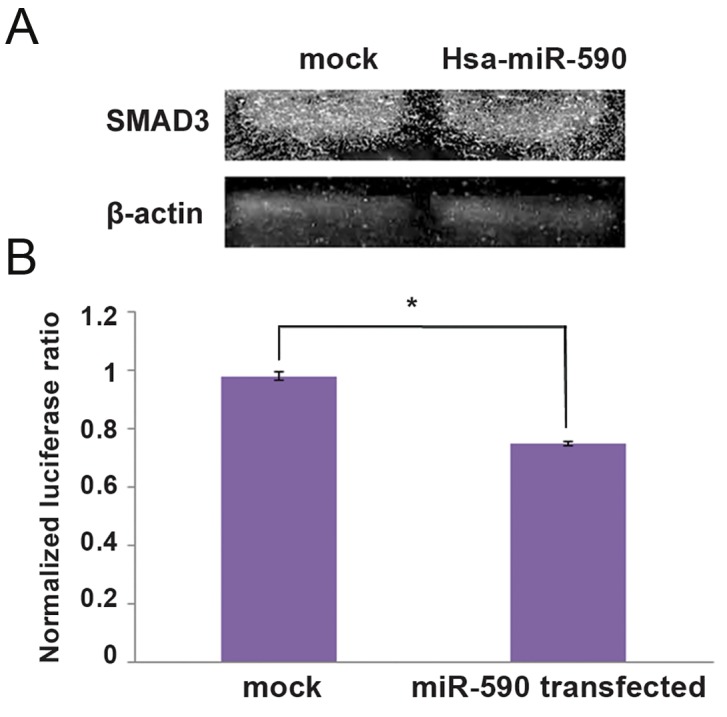
Evidence for interaction of SMAD3 transcripts with hsa-miR-590-5p. A. Down-regulation of SMAD3 expression following hsamiR- 590 overexpression detected through western analysis. Bands were quantitated using Total Lab Quant and results indicated 10% down-regulation of SMAD3 protein compared with the mock control and B. Luciferase assay validating SMAD3 as a target of hsa-miR- 590-5p. Luciferase activity was decreased in hsa-miR-590-5p transfected cells more than 15% compared with Hek293t cells co-transfected with mock, which supported direct interaction of hsa-miR-590-5p and the 3´-UTR of SMAD3 (*; P<0.05 vs. mock control).

The effect of miR-590-5p overexpression on SMAD3 expression was assessed by determining the level of SMAD3 using western blotting. This technique demonstrated that in the CDC overexpressing miR-590, SMAD3 was less abundant compared with the mock control (Fig.3A). Direct interaction of miR-590-5p with SMAD3 transcripts was also investigated using dual luciferase assay. The luciferase activity was measure in cells co-transfected with psicheck2-SMAD3-3´-UTR and pLenti-III-miR-590 or pLenti-III-Blank vectors. Compared with the mock controls, co-transfection of psi-check2SMAD3-3´-UTR with pLenti-III-miR590-5p resulted in about 15% decreased luciferase activity in the Hek293t cell line (Fig.3B). 

### Cell cycle effect of miR-590-5p overexpression in HeLa and SW480 cell lines 

**Fig.4:**
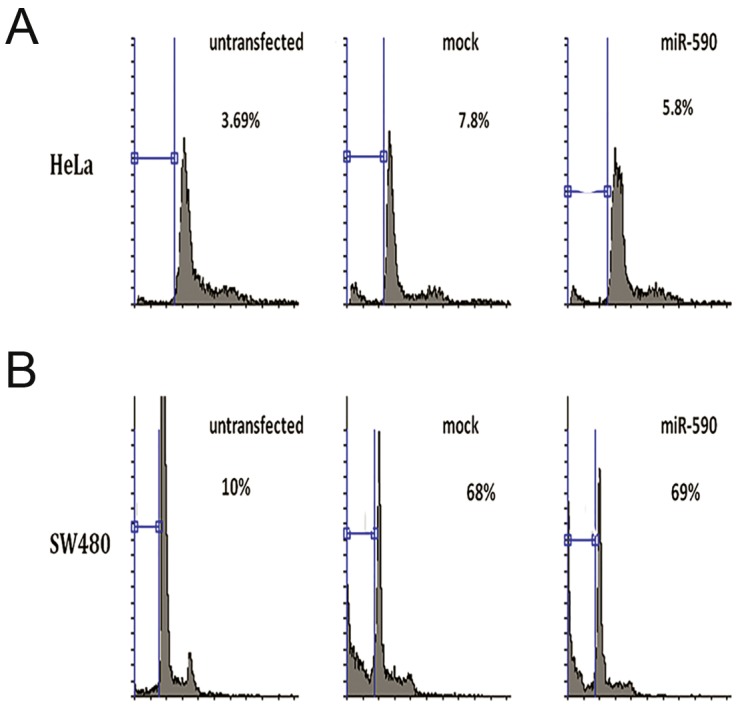
Cell cycle analysis of the cells overexpressing hsa-miR-590-5p. No significant effect of hsa-miR-590 overexpression was observed on cell cycle in A. HeLa and B. SW480 cell lines compared with respective mock controls.

MiR-590-5p was overexpressed in HeLa and SW480 cell lines to analyze its overexpression effect on the cell cycle, using flow cytometry. Results indicated no significant effect on cell cycle compared with the mock-transfected cells (Fig.4). 

## Discussion

SMAD proteins have significant roles in different signaling pathways especially the TGFβ/ BMP signaling pathway (16,17). SMAD3, one of the main players in TGFβ signaling, has different roles in cell proliferation, apoptosis and migration (18,19). As a result, this gene has shown to be critical to cell differentiation and cancer development (20,22). miRNAs are now identified as a key regulator of biological processes (23,25) including the TGFβ signaling pathway and consistently mis-expression of specific miRNAs is detected in many cancer types (26,27). However, the molecular mechanism of TGFβ regulation still remains largely unknown. In previous studies, the overexpression of miR-590-5p in CDC resulted in a significant down-regulation of TGFβR2, TGFβR1 and TGFβ1 target genes (15). In the present study, the regulatory effects of miR-590-5p on the TGFβ signaling pathway and its direct interaction with SMAD3 were also investigated. Preliminary bioinformatics analysis indicated that SMAD3 is a potential target gene of miR590-5-p. We and others have used the luciferase assay as a sensitive genetic reporter system to investigate miRNA-target transcript interaction (28,30). Following successful miR-590-5p overexpression, luciferase activity of co-transfected reporter was decreased about 15%, thus validating the direct interaction of this miRNA with the 3´-UTR sequence of SMAD3 predicted bioinformatically. Next we examined whether this direct interaction affects the protein level production of SMAD3 and consistent with the luciferase assay result, SMAD3 reduction was observed compared with the mock control transfection. This effect is consistent with the model previously presented by Ekhteraei-Tousi et al. (15), suggesting a feedback loop for miR-590-5p against the TGFβ signaling pathway. According to our current results and this model, miR-5905p down-regulates SMAD3 which is a negative regulator of STAT5, a protein that positively regulates miR-590-5p expression. That suggests that miR-590-5p intensifies its own expression through down-regulation of SMAD3. 

Recently, contribution of SMAD3 to G1 arrest was reported in breast cancer (31). This led us to investigate its overexpression effect on cell cycle of cancer cell lines. Flow cytometry results of transfected HeLa and SW480 cell lines showed no significant effect of miR-590-5p overexpression on cell cycle, consistent with another report emphasizing that SMAD3 deficiency may not alter cell cycle progression (32). Knowing about its role in TGFβ signaling, it remains to be tested if the cell cycle effect of hsa-miR-590 could be justified through its effect on SMAD3 alone. 

## Conclusion

We show that miR-590-5p as a regulator of TGFβ signaling pathway, targets the 3´-UTR of SMAD3 and reduces its expression. By reducing SMAD3 protein level as the main member of the TGFβ signaling pathway, miR-590-5p may attenuate the final effect of this pathway. 
